# Annotations

**Published:** 1890-05-24

**Authors:** 


### Annotations.

**A Sanitary Duel at Sheffield.**

The town of Sheffield, which, perhaps, surpasses any other in England in smoke and grime and blackness, has two public bodies devoted to sanitation and municipal soap and water. The more ancient and responsible body is the Health Committee of the Town Council; the younger and apparently more energetic organization is the Association for Promoting Sanitary Reform and the Better Housing of the Poor. A few days ago the latter body met in the Cutlers' Hall, and a very representative meeting, consisting of aldermen, clergymen, and members of other professions, under the presidency of the Venerable Archdeacon Blakeney, came together. Those who have had experience of voluntary associations like that at Sheffield know that usually talk preponderates over work at their meetings in a very decided degree. At this particular meeting, however, one member, Mr. Jonathan Taylor, was evidently resolved that his talk should lead to some result, even if it did not clear away for ever the melancholy pall of Sheffield smoke. Mr. Taylor took up his parable against insanitary houses. He related the case of a man who had resorted to the extraordinary course of leaving his dwelling and erecting a tent on some unoccupied ground. The would-be Arab, Mr. Taylor said, had been sent to prison for eighteen days. No doubt he deserved it, continued Mr. Taylor; but what about the owner of the house: ought not he to have had eighteen days? And should not the Medical Officer of Health, and the Mayor, and the Chairman of the Health Committee each have had eighteen days in turn? The Chairman of the Health Committee, Mr. Alderman Clegg, got into a towering rage at Mr. Taylor's pleasantries about imprisonment. It was grossly unfair, he considered, to make such statements about the Medical Officer of Health in his absence; and if he, Alderman Clegg, was to be attacked in this way, he maintained that he ought to have had notice beforehand. He was a subscriber to this very society and a member of the Town Council, and gave both time and money for the benefit of the community. And yet they were to be talked of in the way that Mr. Taylor had adopted; it was simply abominable. Those little amenities of Sheffield public life seem to be not uncommon. The other members of the association present laughed heartily, first with Mr. Taylor and then at Mr. Alderman Clegg. Meanwhile, Sheffield is exactly where it was before—black, sulphurous, horrible! It and other towns like it are not only a disgrace to England but to civilisation itself. Smoke can be consumed, and in the interests of the public, as opposed to those of manufacturers, it ought to be consumed. Alderman Clegg considers that Sheffield needs more smoke rather than less—smoke being the measure of trade. Nothing of the kind, Mr. Alderman! The community at large would be both happier and better if there were much less of some kinds of trade, and if workers were compelled to return to the cultivation of the land. That man is the sorriest and stupidest of all God's creatures who thinks that the beginning and end of human activity is to make money. Sheffield, with other towns of the same kind, is full of such men.

**Is She Not a Nurse?**

Sick nursing, it is to be feared, is going to prove an unhealthy occupation. It is a very common thing nowadays to meet nurses in the street, and the observant doctor cannot but notice how pallid and anæmic many of them look. No one need be surprised that large numbers of nurses are so pale and bloodless. Their life is for the most part spent indoors; and indoor life is bound to produce anæmia sooner or later. Moreover, the work of a nurse is anxious, responsible, solitary. She eats all her meals alone, or in the presence of a sick person. She is not at home in the kitchen, and she is seldom welcomed as a friend or a member of the family upstairs. She has long hours of work, short periods of relaxation, and is often disturbed in her sleep. Many employers look upon her as a kind of eight-day clock, which, if periodically wound up, ought to "go" for ever. How can she be tired; is she not a nurse? How can she have a poor appetite; is she not a nurse? Why should she have a headache, and be depressed in spirits after a sleepless night; is she not a nurse? There are hundreds of people who seem to think that because a woman is a nurse, therefore, she is no longer a woman, but a kind of cast-iron machine, or, as we have said, an eight-day clock, wound up, and, by the nature of the case, warranted to "go." To say that such views are stupid is to say little; they are inhuman. The Bishop of Ripon, Dr. Boyd Carpenter, struck the right note at Lady George Hamilton's meeting the other day, when he said he "looked forward to the time when it would be in the power of any doctor to say at once to anyone of these nurses who worked so hard and ran such risks: You have had enough nursing for the present; there is an order for you to get that rest which I know you need." What is undoubtedly now required is ready and sufficient means of securing rest for a nurse before she is broken down and ill. That she will break down and be ill is certain if she keep on long enough with her work without rest. That she cannot afford to rest, if at the same time she has to pay for her own lodging and food, is equally certain. Proper provision for trained nurses during convalescence and holidays is one of the most urgent wants of the times. Princess Christian, the Duchess of Abercorn, Sir James Paget, and others, have undertaken an altogether admirable work in raising funds, but we would counsel them to use their money to send nurses to suitable places, and not to build any special home which would not meet the needs of the case. Fifteen thousand pounds is needed; and if all the friends of nurses, and nurses themselves, will make a united and generous effort, the money will soon be forthcoming.

**A Summer Duty.**

The rich and fashionable people of the West-end take a genuine interest in the children of the poor. At Devonshire House, a few days ago, a very large number of them met together to promote the interests of the Children's Country Holiday Fund. They have begun their work in good time, with the very first flush of summer. It is only six years since the Country Holiday Fund was started, and last year it spent more than thirteen thousand pounds. This year we hope the thirteen thousand will become thirty thousand, for neither in hospitals nor in any other way can money be put to more serviceable use than by sending the pale and spiritless children of the poorer parts of large towns to find life for the body and delight for the mind in the fields and woods of the country. Will those who have money to spare consider three or four leading facts about these children's country holidays? First of all, the Fund is administered entirely, or almost entirely, by unpaid workers. It has thirty-six local committees, each doing its very utmost to get the last pennyworth of value out of the contributed money. There are in connection with it in London five hundred voluntary workers, and in the villages there are other five hundred persons whose work is also voluntary, and who superintend the housing and general comfort of the children. Last summer nearly twenty-one thousand children were sent to the country for a fortnight each, and the cost of each child's fortnight, including railway fares, food, and lodging was only about fourteen shillings. Of that sum the parents paid about a third. It follows, then, that everybody who gave the modest contribution of ten shillings to the Fund had the rare privilege of sending one child and a fraction of another to a delightful country village for a fortnight. Does the London season offer a single pleasure so keen and wholesome as this for the money? It only remains to add that the address of the Fund is at 10, Buckingham Street, Strand. Perhaps we may also say, in these times of ecclesiastical strife, that it is quite unsectarian.

